# A model for predicting bacteremia in patients with community-acquired pneumococcal pneumonia: a retrospective observational study

**DOI:** 10.1186/s12890-018-0572-1

**Published:** 2018-01-30

**Authors:** Yasuyoshi Washio, Akihiro Ito, Shogo Kumagai, Tadashi Ishida, Akio Yamazaki

**Affiliations:** 1 Department of Respiratory Medicine, Saiseikai Fukuoka General Hospital, 1-3-46 Tenjin, Chuo-ku, Fukuoka, 810-0001 Japan; 2 Department of Respiratory Medicine, Ohara Memorial Kurashiki Healthcare Foundation, Kurashiki Central Hospital, 1-1-1 miwa, Kurashiki, Okayama 710-8602 Japan

**Keywords:** Bacteremia, Blood culture, Community-acquired pneumonia, Pneumococcal pneumonia

## Abstract

**Background:**

Pneumococcal pneumonia causes high morbidity and mortality among adults. This study aimed to identify risk factors for bacteremic pneumococcal pneumonia, and to construct a prediction model for the development of bacteremia in patients with community-acquired pneumococcal pneumonia.

**Methods:**

We retrospectively analyzed data from patients hospitalized with community-acquired pneumococcal pneumonia between April 2007 and August 2015. Logistic regression models were applied to detect risk factors for pneumococcal bacteremia, and a receiver operating characteristic curve was used to devise a prediction model.

**Results:**

Based on the results of sputum cultures, urine antigen tests, and/or blood cultures, 389 patients were diagnosed with pneumococcal pneumonia, 46 of whom had bacteremia. In the multivariate analysis, age < 65 years, serum albumin level < 3.0 g/dL, need for intensive respiratory or vasopressor support (IRVS), and C-reactive protein level > 20 mg/dL were identified as independent risk factors for the development of pneumococcal bacteremia. The bacteremia prediction score based on receiver operating characteristic curve analysis had a sensitivity of 0.74 and a specificity of 0.78 in patients with two risk factors. The area under the receiver operating characteristic curve was 0.77 (95% confidence interval (CI), 0.70–0.85).

**Conclusions:**

Age < 65 years, hypoalbuminemia, IRVS, and high C-reactive protein level on admission are independent risk factors for the development of bacteremia in patients with community-acquired pneumococcal pneumonia. A prediction model based on these four risk factors could help to identify patients with community-acquired pneumococcal pneumonia at high risk of developing bacteremia; this can be used to guide antibiotic choices.

**Trial registration:**

UMIN-CTR UMIN 000004353. Registered 7 October 2010. Retrospectively registered.

## Background

*Streptococcus pneumoniae* is the most common bacterial pathogen and the most frequent cause of death in patients with community-acquired pneumonia (CAP) [1–3]. Gram staining of sputum, sputum culture, blood culture, and urinary antigen tests are common methods of identifying bacterial pathogens [4]. Antibiotic treatment is prescribed for hospitalized patients on admission. The choice of antibiotic may be empiric or pathogen-directed; the latter based on the results of sputum Gram staining, urinary antigen testing, and other patient characteristics. Although there is no reported difference in outcome between initial pathogen-directed or empiric broad spectrum antibiotic treatment for patients with CAP, pathogen-directed antibiotic treatment based on Gram staining reduces the cost and overuse of antibiotics [5, 6]. However, obtaining good quality sputum for Gram staining is often difficult, and sputum culture may lack specificity [7]. Blood culture, because of its high specificity [8], is an important means of identifying pathogens causing pneumonia and for predicting clinical outcomes. In patients with CAP, the development of bacteremia or septic shock is associated with increased risk of mortality [9, 10].

Given that blood culture results are usually not available until several days after admission, a model predicting bacteremia would be a useful tool for anticipating a patient’s clinical course and for choosing appropriate antibiotic treatment. Metersky et al. [11] reported an association between bacteremic pneumonia and vital signs, liver disease, and laboratory findings on admission in patients with CAP. Previously reported risks factors for bacteremic pneumococcal CAP are smoking, the use of immunosuppressive drugs, younger age, and the presence of diabetes mellitus [12]. Regarding antibiotic treatment for bacteremic pneumococcal pneumonia, the in-hospital mortality rate is lower in patients treated with a beta-lactam plus a macrolide than in those treated with beta-lactam monotherapy [13].

Despite their usefulness, existing models for predicting bacteremia in patients with CAP can be complicated to apply in the clinical setting, where patient populations and pathogens are non-uniform [14, 15]. Moreover, there are no existing models for predicting bacteremia in pneumococcal CAP, specifically. In this study, we aimed to identify risk factors associated with bacteremia and to construct a model to predict bacteremia in patients with pneumococcal CAP. Such a model may help clinicians to decide on the most appropriate antibiotic treatment to initiate on admission, including whether or not to use combination therapy.

## Methods

### Study design and setting

We retrospectively analyzed the data of all patients hospitalized for pneumococcal CAP and enrolled in a prospective observational cohort study at Kurashiki Central Hospital, Okayama, Japan, between April 2007 and August 2015. Pneumonia was diagnosed based radiographic findings (new infiltrates compatible with a diagnosis of pneumonia on chest x-ray) and clinical findings (acute-onset clinical symptoms suggestive of a lower respiratory tract infection, such as cough, sputum production, fever, pleural chest pain, or dyspnea). Chest x-ray images demonstrating new infiltrates were assessed by two or more respiratory medicine clinicians. Patients aged ≤ 15 years and those with immunosuppression or hospital-acquired or healthcare-associated pneumonia, were excluded. Included patients were divided into two groups; one with bacteremia and the other without. This study was approved by the institutional review board of Kurashiki Central Hospital (approval number 2235). Based on the Ethical Guidelines for Medical and Health Research Involving Human Subjects of the Ministry of Health, Labour and Welfare, we notify the research subjects of, or make public, information concerning the research in our hospital. All patients gave their informed consent to participate in this study by being given opportunities to refuse to participate.

### Clinical characteristics

The severity of pneumonia was assessed for all patients on admission using the CURB-65 score (a severity score based on confusion, blood urea nitrogen (BUN) levels, respiratory rate, blood pressure, and age), the Pneumonia Severity Index score, or the Infectious Diseases Society of America (IDSA)/American Thoracic Society (ATS) criteria for severe CAP [16–18].

The following patient data were recorded: age, sex, smoking status, high alcohol consumption (defined as consuming ≥5 alcoholic drinks per day for >10 years), multilobar pneumonia, previous antibiotic treatment, corticosteroid use before hospitalization, Eastern Cooperative Oncology Group (ECOG) performance status score (0: *normal activity*, 1: *some symptoms, but no bed rest during daytime*, 2: *bed rest for less than 50% of daytime*, 3: *bed rest for more than 50% of daytime*, 4: *unable to get out of bed*), and underlying diseases (chronic obstructive pulmonary disease, bronchial asthma, chronic cardiovascular disease, cerebrovascular disease, chronic renal disease, chronic liver disease, malignant disease, and/or diabetes mellitus). In addition, vital signs at the time of arrival, laboratory findings (concentrations of blood glucose and BUN; serum albumin, C-reactive protein (CRP), and sodium concentrations; hematocrit; and partial pressure arterial oxygen/fraction of inspired oxygen (PaO2/FiO2) ratio) and diagnostic methods, were recorded. We recorded admission intensive care unit (ICU), vasopressor drug use, mechanical ventilation, and intensive respiratory or vasopressor support (IRVS; i.e., invasive or noninvasive mechanical ventilation or infusion of vasopressors for blood pressure support [19]).

Pneumococcal CAP was defined as at least one positive result for *Streptococcus pneumoniae* on blood, sputum, tracheal bronchial aspirate, and/or urinary antigen testing using the BinaxNOW® Streptococcus pneumoniae Antigen Card (Alere Inc., Waltham, MA, USA) in a patient with clinical and radiographic features of CAP. Sputum specimens with >25 leucocytes per field were considered of sufficient quality for diagnosis. Bacteremic pneumococcal pneumonia was diagnosed on the basis of isolating *S. pneumoniae* from blood cultures obtained before the parenteral administration of antibiotics.

### Analysis

Fisher’s exact test and the non-parametric Mann-Whitney *U* test were used to detect significant differences in categorical and continuous variables, respectively, between the two groups. Univariate and multivariate logistic regression analyses were performed to identify variables predictive of pneumococcal bacteremia development. Variables with a *p*-value < 0.05 in the univariate analysis were included in the multivariate logistic regression models using a stepwise approach. Cut-off values for these variables were obtained from previous studies [11, 15, 18, 20, 21]. Receiver operating characteristic (ROC) curves were constructed to predict the development of pneumococcal bacteremia based on factors detected in the multivariate analysis. All statistical tests were two-tailed, and a *p*-value < 0.05 was considered significant. Analyses were conducted using R statistical software (version 3.4.1, Vienna, Austria).

## Results

Of 1829 patients hospitalized with CAP during the study period, 389 (21.3%) were diagnosed with pneumococcal pneumonia. Of these, 46 (12%) had concomitant bacteremia (bacteremic group) and 343 (88%) did not (non-bacteremic group). The baseline characteristics of both groups are listed in Table 1. The following were found more frequently in the bacteremic than in the non-bacteremic group: Age < 65 years, vasopressor use, mechanical ventilation, IRVS, and an increased number of ATS/IDSA severe criteria.

**Table 1:** Characteristics of patients hospitalized with bacteremic and non-bacteremic community-acquired pneumococcal pneumonia

Characteristic	Category	No bacteremia (*n* = 343)	Bacteremia (*n* = 46)	*p* value
Demographic characteristics	Age (years), median (range)	77 (20–97)	68 (29–91)	0.001^a^
	Female sex	119 (34.7)	15 (32.6)	0.87
	Current smoker	53 (15.5)	9 (19.6)	0.52
	High alcohol consumption	9 (2.6)	3 (6.5)	0.16
Underlying disease(s)	Chronic cardiovascular disease	92 (26.8)	8 (17.4)	0.21
	COPD	86 (25.1)	10 (21.7)	0.72
	Bronchial asthma	62 (18.1)	6 (13.0)	0.54
	Diabetes mellitus	53 (15.5)	7 (15.2)	1.00
	Chronic liver disease	17 (5.0)	3 (6.5)	0.79
	Chronic renal disease	27 (7.9)	3 (6.5)	1.00
	Malignant disease	33 (9.6)	5 (10.9)	0.79
	Cerebrovascular disease	70 (20.4)	6 (13.0)	0.32
	Previous antibiotic treatment	69 (20.1)	6 (13.0)	0.32
Corticosteroid use before hospitalization	Yes	3 (0.9)	0 (0.0)	1.00
Clinical characteristics on admission	Temperature (°C)	38.2 (34.4–40.9)	38.1 (34.3–40.0)	0.63
	Respiratory rate (breaths·min^−1^)	24 (10–60)	26 (10–50)	0.005^a^
	Heart rate (beats·min^−1^)	100 (52–173)	109 (78–163)	0.002^a^
	Systolic blood pressure (mmHg)	128 (30–226)	123 (65–191)	0.36
	Impaired consciousness	63 (18.4)	9 (19.6)	0.84
	Multilobar pneumonia	209 (60.9)	33 (71.7)	0.20
Laboratory findings	Serum albumin (mg·dL^−1^)	3.4 (1.5–4.8)	2.7 (0.5–4.3)	<0.001^a^
	BUN (mg·dL^−1^)	20 (4–161)	26 (11–105)	0.002^a^
	Blood glucose (mg·dL^−1^)	133 (66–517)	130 (61–413)	0.76
	Serum CRP (mg·L^−1^)	12.5 (0.07–48.6)	24.9 (0.55–51.1)	<0.001^a^
	Hematocrit (%)	37.6 (20.3–54.2)	36.0 (25.0–51.9)	0.67
	Sodium (mEq·L^−1^)	137 (111–150)	135 (129–146)	0.03^a^
	P/F ratio	248 (35.7–512)	197 (35.7–420)	0.01^a^
Microbiologic diagnosis	Sputum Gram stain	89 (25.9)	12 (26.7)	1.00
	Sputum culture	244 (71.1)	32 (69.6)	0.86
	Urinary antigen test	200 (61.0)	37 (76.1)	0.05
	Sputum culture and urinary antigen test	109 (31.8)	21 (45.7)	0.07
ECOG performance status	0	54 (21.2)	12 (46.2)	0.07
	1	112 (43.9)	10 (38.5)	
	2	50 (19.6)	2 (7.7)	
	3	24 (9.4)	2 (7.7)	
	4	15 (5.9)	0 (0)	
CURB-65 class	0	32 (9.3)	4 (8.9)	0.12
	1	86 (25.1)	10 (22.2)	
	2	127 (37.0)	10 (22.2)	
	3	67 (19.5)	14 (31.1)	
	4	26 (7.6)	5 (11.1)	
	5	5 (1.5)	2 (4.4)	
PSI class	I	7 (2.0)	1 (2.2)	0.37
	II	34 (9.9)	5 (10.9)	
	III	80 (23.4)	12 (26.1)	
	IV	150 (43.9)	14 (30.4)	
	V	71 (20.8)	14 (20.4)	
IDSA/ATS severe		120 (35.0)	29 (63.0)	< 0.001^a^
ICU admission	Yes	16 (4.7)	11 (23.9)	< 0.001^a^
Vasopressor use	Yes	14 (4.1)	10 (21.7)	< 0.001^a^
Invasive or noninvasive mechanical ventilation	Yes	18 (5.2)	12 (26.1)	< 0.001^a^
IRVS	Yes	20 (5.8)	13 (28.3)	< 0.001^a^

^a^denotes significant value

*Abbreviations*: *COPD* chronic obstructive pulmonary disease, *IRVS* intensive respiratory or vasopressor support, *ICU* intensive care unit, *ECOG* Eastern Cooperative Oncology Group, *CURB-65* confusion, urea > 7 mmol·L^−1^, respiratory rate ≥ 30 breaths·min^−1^, low blood pressure (systolic < 90 mmHg or diastolic ≤ 60 mmHg) and age ≥ 65 y, *PSI* Pneumonia Severity Index, *IDSA* Infectious Diseases Society of America, *ATS* American Thoracic Society, *BUN* blood urea nitrogen, *CRP* C-reactive protein, *P/F ratio* PaO_2_/FiO_2_ ratio

Patients in the bacteremic group also had higher respiratory and heart rates, lower serum albumin levels, higher BUN and CRP levels, lower serum sodium concentrations, and lower PaO2/FiO2 ratios. There were no significant differences between the two groups regarding underlying disease, sex, smoking status, high alcohol consumption, previous antibiotic treatment, corticosteroid treatment before hospitalization, multilobar pneumonia, level of consciousness, method of diagnosis of pneumococcal pneumonia, ECOG performance status, or pneumonia severity indices.

**Fig. 1:**
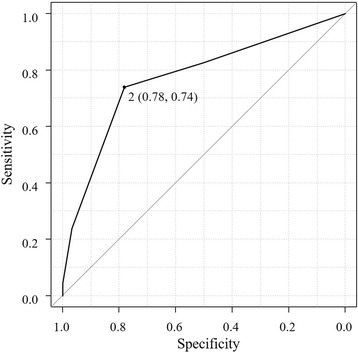
Receiver-operating characteristic curve for four factors predictive of bacteremia in community-acquired pneumococcal pneumonia patients. Area under the curve = 0.77 (95% confidence interval, 0.70–0.85). The four factors predictive of bacteremia were age < 65 years, albumin level ˂3.0 g/dL, need for intensive respiratory or vasopressor support, and C-reactive protein level > 20 mg/dL. Each factor was allocated one point in the prediction model. For patients scoring two points, the specificity was 0.78 and sensitivity was 0.74

**Fig. 2:**
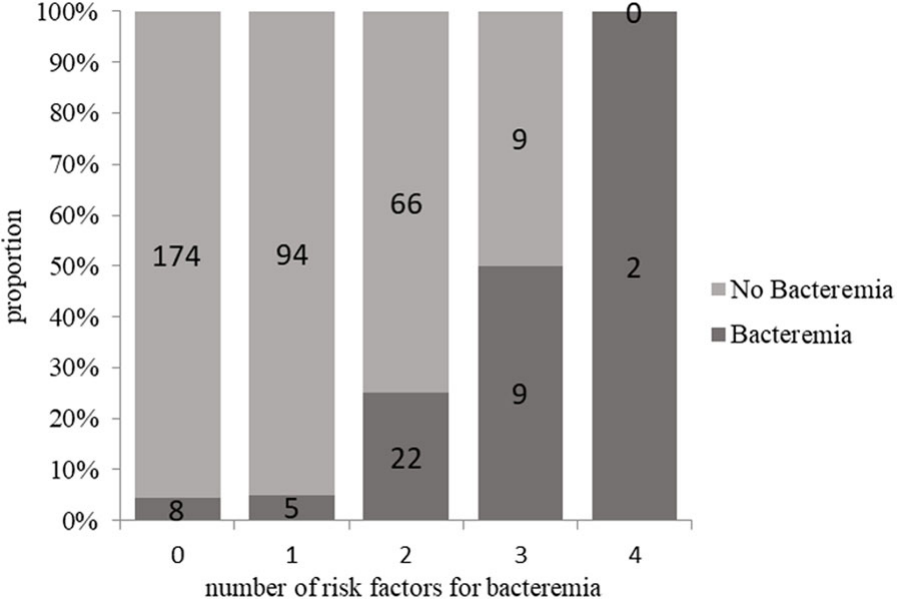
Proportion of patients with community-acquired pneumococcal pneumonia according to bacteremia prediction model score. Four significant risk factors for bacteremia in community-acquired pneumococcal pneumonia patients were identified (age < 65 years, albumin level < 3.0 g/dL, need for intensive respiratory or vasopressor support, and C-reactive protein level > 20 mg/dL). Each risk factor was allocated one point in the bacteremia prediction model. The figure shows the proportion of patients with 0, 1, 2, 3, and 4 risk factors with and without bacteremia

The univariate logistic regression analysis revealed that age < 65 years, respiratory rate > 30 breaths/min, heart rate > 125 beats/min, IRVS, albumin < 3.0 mg/dL, BUN > 30 mg/dL, serum sodium < 130 mEq/L, and CRP > 20 mg/dL were associated with increased odds of pneumococcal bacteremia. The multivariate analysis identified age < 65 years, IRVS, albumin < 3.0 mg/dL, and CRP > 20 mg/dL as being independent risk factors for pneumococcal bacteremia (Table 2). We devised a predictive score using these four factors, allocating one point to each factor. The area under the ROC curve was 0.77 (95% confidence interval (CI), 0.70–0.85) with 0.74 sensitivity and 0.78 specificity for patients with two points (Fig. 1). Only 5% (13/277) of patients with 0 or 1 point had bacteremia, whereas all patients with 4 points had bacteremia (Fig. 2, Table 3).

**Table 2:** Univariate and multivariate analyses showing potential risk factors for bacteremia in community-acquired pneumococcal pneumonia

Variable	Univariate analysis		Multivariate analysis	
	OR (95% CI)	*p*-value	OR (95% CI)	*p*-value
Age < 65 years	3.00 (1.55–5.83)	0.006^a^	3.64 (1.73–7.67)	< 0.001^a^
Respiratory rate > 30 breaths·min^−1^	2.65 (1.39–5.03)	0.003^a^		
Heart rate > 125 beats·min^−1^	2.44 (1.12–5.35)	0.03^a^		
IRVS	6.36 (2.90–13.9)	< 0.001^a^	3.99 (1.71–9.33)	0.001^a^
Serum albumin < 3.0 mg·dL^−1^	4.18 (2.21–7.92)	< 0.001^a^	3.35 (1.63–6.87)	0.001^a^
BUN >30 mg·dL^−1^	1.87 (0.97–3.57)	0.06		
Serum sodium < 130 mEq·L^−1^	3.10 (1.05–9.13)	0.04^a^		
P/F ratio < 250	2.57 (1.36–4.85)	0.004^a^		
Serum CRP > 20 mg·dL^−1^	4.12 (2.18–7.77)	< 0.001^a^	2.39 (1.21–4.75)	0.01^a^

^a^denotes significant value

*Abbreviations*: *OR* odds ratio, *CI* confidence interval, *BUN* blood urea nitrogen, *P/F ratio* PaO_2_/FiO_2_ ratio, *CRP* C-reactive protein, *IRVS* intensive respiratory or vasopressor support

**Table 3:** Accuracy of the diagnostic score according to the number of risk factors for bacteremia

Number of risk factors	Bacteremia	No Bacteremia	Sensitivity	Specificity
0	8 (17.4%)	174 (50.7%)	1.00	–
≥ 1	38 (82.6%)	169 (49.3%)	0.83	0.50
≥ 2	33 (71.7%)	75 (21.9%)	0.74	0.78
≥ 3	11 (23.9%)	9 (2.6%)	0.24	0.97
4	2 (4.3%)	0	0.043	1.00

## Discussion

The main finding of this study is that age < 65 years, IRVS, hypoalbuminemia, and elevated CRP levels were identified as risk factors for pneumococcal bacteremia in patients with pneumococcal CAP. Our prediction model using these four risk factors is the first system exclusively designed to identify pneumococcal bacteremia in patients with pneumococcal CAP. Previous studies investigating bacteremia in patients with pneumonia identified different risk factors. This discrepancy is due to the heterogeneity in causative pathogens or study cohorts; for example, some studies included patients with pneumonia irrespective of etiology while others included only patients with CAP [9, 10, 12, 20, 22, 23]. Our study is important as our sample was limited to patients with pneumococcal CAP. Although Amaro et al. [21] reported on risk factors for bacteremia in patients with pneumococcal CAP, there is still a paucity of information on this topic, and there is currently no specific prediction model.

Younger age has been reported to be a significant and independent risk factor for bacteremia in patients with CAP [12, 20, 24]; our findings concur with this. However, it is not clear why younger patients are at greater risk of bacteremia; pneumococcal vaccination and the serotype of *S. pneumoniae* may play a role. In Japan, the 13-valent pneumococcal conjugate vaccine was approved for use as a voluntary vaccine for adults aged ≥ 65 years in June 2014. The 23-valent pneumococcal polysaccharide vaccine was approved for use in 1988 and routine immunization for those aged ≥ 65 years was introduced in October 2014. These pneumococcal vaccines are recommended for adults aged ≥ 65 years to prevent pneumonia and other invasive pneumococcal diseases, and to improve clinical outcomes [25, 26]. Because the rate of vaccination increases depending on age and younger adults usually don’t take pneumococcal vaccines, this may have led to younger age being identified as a risk factor for bacteremia [27]. Moreover, *S. pneumoniae* serotype 1 is the most commonly isolated serotype in invasive pneumococcal disease; it is more likely than the other serotypes to be identified in young patients without comorbidities [28]. The results from this study suggest a relationship between the pneumococcal serotype and study population with bacteremia; however, a limitation of our study was that we did not collect information on serotype, so this relationship could not be assessed.

Previous studies have shown low serum albumin or high serum CRP concentrations to be risk factors for bacteremia in patients with CAP [15, 20, 21, 29]; our findings are consistent with this. Low serum albumin concentration is a risk factor for and a predictor of morbidity and mortality, regardless of the disease [30], and a relationship exists between hypoalbuminemia and severe infection due to the elevation of cytokine levels during systemic inflammation [31]. Hypoalbuminemia, an elevated inflammatory response, or both, results in worse clinical outcomes for patients with severe infection.

Capelastegui et al. [10] reported that patients with pneumococcal bacteremia had a higher frequency of requiring mechanical ventilation and treatment for septic shock with vasopressor drugs than did non-bacteremic patients, but there was no difference in the rate of ICU admission. In contrast, in the present study, a greater proportion of patients in the bacteremic group than in the non-bacteremic group required vasopressors, mechanical ventilation, and ICU admission. This discrepancy may be due to differences in the criteria used for ICU admission between hospitals and countries. Then we chose the need for IRVS—and for vasopressor drugs, in particular—, which is a more objective marker of CAP severity than is simple ICU admission [19]. Although previous studies have reported that low systolic blood pressure is a risk factor for bacteremia [20, 24], this was not identified as a significant risk factor in the present study; however, the use of vasopressors was. This may have been influenced by clinicians’ choice of vasopressors that provide direct β1 stimulation (such as dobutamine) for cardiac dysfunction in patients with invasive pneumococcal disease.

Patients with pneumococcal pneumonia or sepsis are at increased risk of a concurrent acute cardiac event (myocardial infarction, arrhythmia, or new or worsening congestive heart failure caused by suppression of ventricular function) due to the severe inflammatory response and elevated levels of cytokines triggered by the use of antibiotics, which rapidly increases the release of bacterial cell wall fragments into the blood stream [32, 33]. In vitro, platelet activating factor receptors facilitate the binding of circulating *S. pneumoniae* bacterial cell walls to endothelial cells, bacterial entry into organs, and specific uptake into non-phagocytic cells such as cardiomyocytes [34]. In a non-human primate model, Reyes et al. [35] reported that *S. pneumoniae* can invade the blood stream and the myocardium, inducing severe cardiac injury with necroptosis and apoptosis, and disrupting cardiac function; notably, such injury occurred despite the use of antibiotics.

Previous studies involving patients with bacteremic pneumococcal CAP have considered prediction models relating to CAP [11, 15, 20]. Our prediction model is the first and only available model to predict pneumococcal bacteremia specifically. In our study, very few patients who scored 0 or 1 point developed pneumococcal bacteremia, whereas all patients who scored 4 points did. This prediction model can help clinicians to decide on the best antibiotic choice. Regarding treatment, combination therapy with a beta-lactam plus a macrolide is prescribed empirically for patients with CAP because of the possibility of infection with an atypical pathogen. Combination therapy is contraindicated in some patients because of the adverse effects of macrolides, including arrhythmias, and the possibility that frequent use of macrolides will lead to antibiotic resistance. It is unclear whether this combination is useful for all patients with CAP. Douwe et al. [36] reported no difference in 90-day mortality between patients with CAP treated with a beta-lactam alone, a beta-lactam plus a macrolide, or quinolone alone. However, some studies have reported that combination therapy does improve outcomes for patients with bacteremic pneumococcal CAP or for severely ill patients with pneumococcal bacteremia [13, 37]. Clinicians need to choose whether to use combination therapy days before blood culture results are available. By using our prediction model to identify patients (diagnosed with pneumococcal CAP by Gram stain or urinary antigen testing) at high risk of developing bacteremia, the beneficial effects of combination therapy could be maximized—thereby improving outcomes—while minimizing adverse events.

This study has several limitations. First, it was a small, retrospective study, and all patients were selected from a single treatment center. Second, although vaccination against *S. pneumoniae* may reduce the severity of pneumococcal infection or invasive disease and the *S. pneumoniae* serotype may have influenced the occurrence of bacteremia in this study population, neither vaccination status nor *S. pneumoniae* serotype were included as potential risk factors in our analysis. Hence, further cohort studies are needed to verify potential risk factors and validate the performance of our model for predicting pneumococcal bacteremia.

Notwithstanding these limitations, our prediction model is based on easily identifiable risk factors, is simple to use, and provides an effective method for clinicians to identify patients at risk of developing bacteremia.

## Conclusions

Our findings indicate that age < 65 years, hypoalbuminemia, need for IVRS, and high serum CRP levels on admission are independent risk factors for the development of bacteremia in patients with pneumococcal CAP. Our prediction model, based on these four risk factors, may be helpful in treatment guiding decisions—specifically whether initial antibiotic therapy should include combination antibiotic therapy with a beta-lactam plus a macrolide, which is associated with better outcomes in this population.

## Abbreviations

ATS: American Thoracic Society
BUN: Blood urea nitrogen
CAP: Community-acquired pneumonia
CRP: C-reactive protein
ECOG: Eastern Cooperative Oncology Group
IDSA: Infectious Diseases Society of America
PSI: Pneumonia Severity Index
ROC: Receiver operating characteristic
